# Differences in Levodopa Response for Progressive and Non-Progressive Micrographia in Parkinson's Disease

**DOI:** 10.3389/fneur.2021.665112

**Published:** 2021-05-11

**Authors:** Poonam Zham, Sridhar A. Poosapadi, Peter Kempster, Sanjay Raghav, Kanae J. Nagao, Kitty Wong, Dinesh Kumar

**Affiliations:** ^1^School of Engineering, RMIT University, Melbourne, VIC, Australia; ^2^IBM Research Australia, Southbank, VIC, Australia; ^3^Center for Human Movement Research and Analysis, Department of Electronics and Instrumentation, SRM Institute of Science and Technology, Chennai, India; ^4^Department of Medicine, Monash University, Clayton, VIC, Australia; ^5^Neurosciences Department, Monash Medical Centre, Melbourne, VIC, Australia

**Keywords:** Parkinson's disease, levodopa, kinematic, micrographia, dysgraphia

## Abstract

**Background:** Micrographia, one element of the dysgraphia of Parkinson's disease (PD), may be classified according to the presence or absence of a decremental pattern. The decremental form, progressive micrographia, is an expression of the sequence effect seen generally in bradykinesia. Its responsiveness to levodopa has not been evaluated kinematically.

**Objectives:** Aim of this study is to investigate the difference in levodopa response for progressive and non-progressive micrographia.

**Methods:** Twenty-four PD patients and 24 age-matched repeatedly wrote the letter e on a computerized digital tablet. PD patients performed the task two times, in a defined off state and again after levodopa. Scripts were classified as progressive micrographia (PD_PM_) or non-progressive micrographia (PD_NPM_) depending on whether a 10% decrement was seen between the first and final characters of a line of lettering.

**Results:** While levodopa produced a similar response on the MDS-UPDRS motor scale for the two groups, the effect on the two types of micrographia was different. While writing speed improved significantly in both groups after levodopa, the responses were over twofold greater for PD_NPM._ Moreover, the decremental features of PD_PM_–in size, speed, and pen-pressure—were largely unaltered by a levodopa dose.

**Conclusions:** Progressive micrographia is less responsive to levodopa. Our findings agree with research showing that the sequence effect of bradykinesia is relatively resistant to medication. Yet we did not find a weaker overall levodopa motor benefit. Caution is needed in the interpretation of such micrographia measurements for estimating drug responses.

## Introduction

Impairment of handwriting is commonly present in Parkinson's disease (PD), and may predate other symptoms (1). Although reduced size is the most recognized feature, the use of computerized graphic tablets shows that parkinsonian dysgraphia comprises defects in amplitude, velocity, and fluency (2–4). Kinnear Wilson observed that the script size of parkinsonian subjects often trailed off as they continued to write. He proposed a subdivision into progressive micrographia, where there was decrement of letter size across a line of text, and consistent micrographia, where the size of letters was evenly reduced (5).

Other authors have used this classification (6, 7). Wu et al. (8) found that the two types of parkinsonian micrographia show different patterns of activation of the motor system on functional magnetic resonance scans. There are, however, difficulties with definitions of consistent micrographia. Kinnear Wilson suggested a comparison with pre-morbid calligraphy. The establishment of a ‘typical' historical script size is one limitation, since writing size varies considerably in a normal subject depending on writing context (9). In a previous study, we found that classifications of consistent micrographia based on letter size did not yield a distinct subset (10). Only 4% had a purely consistent pattern, while some PD patients with decrement also fell below the consistent cut-off. We concluded that parkinsonian dysgraphia is better classified according to whether progressive micrographia, defined by a 10% decrement between first five and last five sets of sequential letters, is present (PD_PM_) or absent (PD_NPM_).

Progressive micrographia is an expression of the sequence effect of bradykinesia—progressive reduction in speed and amplitude of repetitive actions. The Queen Square Brain Bank criteria sets a decremental description of bradykinesia at the core of the clinical diagnosis of PD (11). Interestingly, while letter size in progressive supranuclear palsy is smaller than in PD, the decrement effect is less conspicuous (12), suggesting that non-progressive micrographia is more typical of progressive supranuclear palsy.

Levodopa has mixed effects on parkinsonian dysgraphia. Two studies that identified groups with progressive micrographia found that decrement in size was relatively unresponsive to levodopa, although neither examined kinematic aspects of writing (6, 7). Handwriting, by virtue of its overlearned, repetitive nature, lends itself to a kinematic examination of the elusive nature of bradykinesia and the effect of dopaminergic treatment on it (13, 14). While objective handwriting analysis has been suggested as a marker for early detection of PD and for monitoring of subsequent progression, previous research is divided on specific features of the levodopa response.

The aim of this work was to investigate the effect of levodopa on dimensional and kinematic measurements of handwriting in participants with and without progressive micrographia. A control group was included to allow comparison with normal left-to-right writing characteristics.

## Materials and Methods

### Participants

Twenty-four patients diagnosed with PD within the last 10 years were recruited from the Movement Disorders Clinic at Monash Medical Centre. All complied with the Queen Square Brain Bank criteria for idiopathic PD (11). Twenty-four healthy age-matched controls were also recruited as normal writing controls. The presence of any advanced PD clinical milestone—visual hallucinations, frequent falling, cognitive disability, or need for institutional care—was an exclusion criterion (15). Cognition was assessed for all participants using the Montreal Cognitive Assessment (MoCA) (16).

For PD participants, the writing tasks were first performed in a practically defined off state (fasting, with anti-parkinsonian medication withheld for at least 12 h) (17). Subjects' usual morning levodopa dose was then administered, their on state taken as the maximum improvement over the next 30–90 min. Motor function in off and on states was scored by a neurologist on the Movement Disorders Society Unified Parkinson's Disease Rating Scale Part III (MDS-UPDRS-III) (18). Scoring of items 3.4–3.8 was extracted as a measure of dominant upper limb bradykinesia. All PD participants were taking levodopa-containing medication. Seven were using extended release levodopa. Levodopa equivalent daily doses were calculated using standard conversion factors (19). Demographic details, including handedness, of PD and control groups are shown in Tables 1, 2. Table 3 presents clinical information on individual PD subjects. The study was conducted in accordance with the Helsinki Declaration on human experiments (revised 2004) and was approved by the Monash Health and RMIT University Human Research Ethics Committees. All participants in this study gave their written informed consent prior to data recording.

**Table 1:** Demographic and clinical information, PD patients and controls.

**Demographics**	**PD**	**Control group**	***p* value**
Number of Subjects, *n*	24	24	
Age, years	71.6 ± 7.14	69.3 ± 5.74	0.2*^a^*
Gender male, female	13, 11	14, 10	1.0*^b^*
Handedness Right, Left	20, 4	22,2	0.7*^b^*
Disease duration, years	5.0 ± 2.88	-	
Levodopa equivalent daily dosage (mg)	480 ± 296	-	

*Values are mean ± standard deviation, comparison between groups was performed using*

a *Independent t test and*

b *Chi-Square 2-tailed test*.

**Table 2:** Demographic and clinical information for PD_PM_ and PD_NPM_ groups.

**Demographics**	**PD_**PM**_**	**PD_**NPM**_**	***p* values**
Number of Subjects, *n*	16	8	
Age, years	70.94 ± 7.59	73.63 ± 6.23	0.4*^a^*
Gender male, female	10, 6	3, 5	0.35*^b^*
Handedness Right, Left	14, 2	5, 1	0.83*^b^*
Disease duration, years	5.1 ± 2.8	5.3	0.84*^a^*
MDS-UPDRS-III *OFF* [0–132]	28.53 ± 10.33	23.88 ± 7.80	0.28*^a^*
MDS-UPDRS-III *ON* [0–132]	20.25 ± 9.99	18.88 ± 3.53	0.73*^a^*
Dominant upper limb bradykinesia score *OFF* [0–12]	3.88 ± 1.67	3.13 ± 1.46	0.29*^a^*
Dominant upper limb bradykinesia score *ON*[0–12]	3.13 ± 1.89	2.13 ± 1.96	0.24*^a^*

*Values are mean ± standard deviation except for gender and handedness. Comparisons between groups performed using*

a *Independent t-test, and*

b *Mann-Whitney U test*.

**Table 3:** Clinical information on PD participants.

	**Age**	**Gender**	**PD duration (years)**	**levodopa equivalent dose(mg)**	**MDS-UPDRS-III *off***	**MDS-UPDRS-III*on***	**Micrographia classification**
*1*	81	F	8	600	23	15	PD_NPM_
*2*	77	F	5	450	32	26	PD_PM_
*3*	62	M	6	950	47	29	PD_PM_
*4*	82	M	7	750	27	22	PD_PM_
*5*	71	M	1	100	17	15	PD_PM_
*6*	65	M	4	550	37	23	PD_PM_
*7*	70	M	5	600	25	20	PD_PM_
*8*	70	M	1	500	15	10	PD_NPM_
*9*	76	M	3	150	25	15	PD_PM_
*10*	70	F	1.5	300	43	40	PD_PM_
*11*	66	M	5	300	40	37	PD_NPM_
*12*	57	M	7	675	34	18	PD_PM_
*13*	64	F	4	400	24	11	PD_NPM_
*14*	78	M	2	100	35	29	PD_PM_
*15*	79	F	5	550	27	29	PD_NPM_
*16*	64	F	5	300	12	12	PD_PM_
*17*	64	F	6	300	8	4	PD_PM_
*18*	72	M	9	950	33	12	PD_PM_
*19*	66	F	10	620	30	20	PD_PM_
*20*	77	F	8	300	15	11	PD_NPM_
*21*	75	M	1.5	300	25	24	PD_NPM_
*22*	78	M	8	450	25	21	PD_PM_
*23*	83	F	1.5	300	26	18	PD_PM_
*24*	77	F	10	1500	22	14	PD_NPM_

### Data Recording Methods

A digital tablet (Wacom Intuos Pro-Large) with the ink-pen having a pressure sensor was used for the experiments. The advantage of this device was that it gave participants the feeling of conventional pen and paper and was likely to be less defamiliarizing to older subjects. Participants sat on a chair, and the tablet was placed on an adjustable desk. The table height and positioning of the tablet on the table was adjusted according to participant preference.

Customized software was developed to record and analyse data from the tablet. The software registered the x-y coordinates, pen pressure and time stamp at a 133 Hz sampling rate.

### Handwriting Tasks and Computation of Handwriting Features

**Figure 1:**
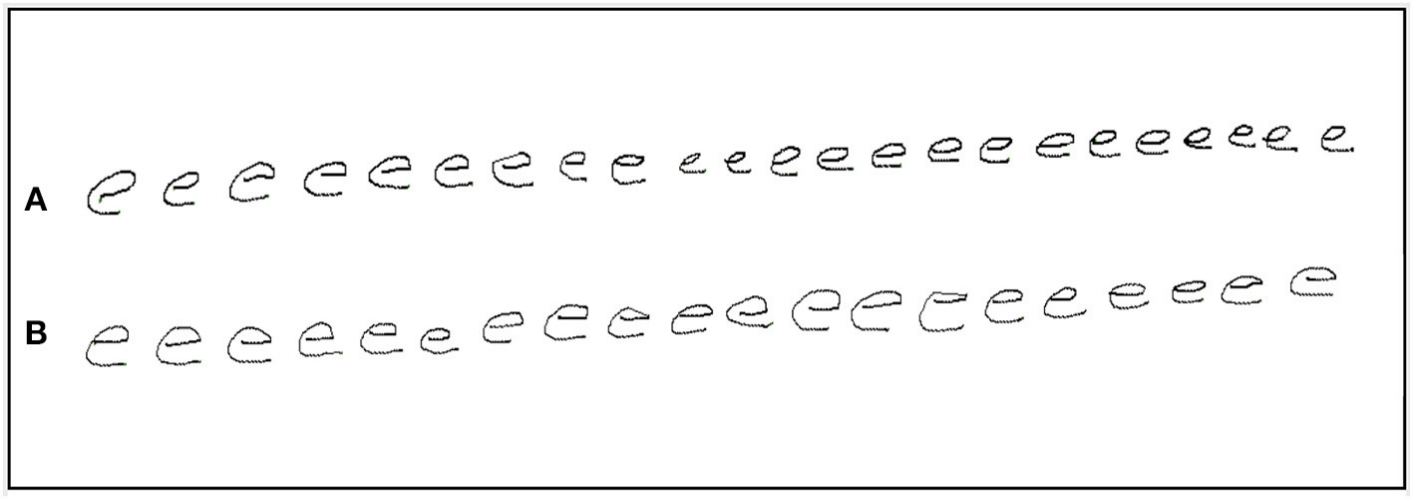
An example of repetition of the letter e by a PD participant with progressive micrographia: off state **(A)** and on state **(B)**.

The task of repeatedly writing of the letter e is routinely used by clinicians to test for micrographia. In this study, the size of each letter e was obtained using x and y coordinates (in mm) and Euclidean formula to calculate stroke length (10). Participants were instructed to write the letter e repeatedly, with pen-up at the end of each letter (see Figure 1). Once 20 repetitions had been exceeded, a researcher gave the instruction to stop writing. Similar protocols have previously been used to study micrographia (10, 20, 21).

The writing data was first segmented based on pen-up and pen-down motions. A threshold of 5 mm was used, and segments of length less than the threshold were excluded. Speed was calculated using a weighted average based on the stroke length measurement of each letter (22).

The initial and final sets of five letters were compared. Progressive micrographia was defined as 10% or greater decrement in the average size of the letter e in the off state (10). PD participants who met this definition were classified as PD_PM_, the remainder as PD_NPM_. Figure 1 shows an example of progressive micrographia.

### Statistical Analysis

Independent sample *t*-test was performed to compare PD and control groups to confirm age-matching, while Chi-Square test was performed for differences in gender and handedness (10). The demographic data was compared between groups using independent *t*-test, while Mann-Whitney U test was used for group differences in gender and right-handedness.

As the data was not normally distributed based on Shapiro-Wilk test, non-parametric Wilcoxon Signed-rank test was performed on related samples from each subject to understand the relationship between the initial and final letter series. Mann-Whitney U test (2-tailed) is suitable for two group analysis hence selected to study the difference between the groups (23).

The sample size of 24 for PD and control groups was determined by a power calculation performed using an online power and sample size calculator developed by Statistical Solutions software (24). The parameters for this were: statistical power = 0.8, with 95% confidence interval and assuming a mean difference between the groups of 110 mm/sec^2^ and pooled standard deviation of 140 (10).

## Results

**Figure 2:**
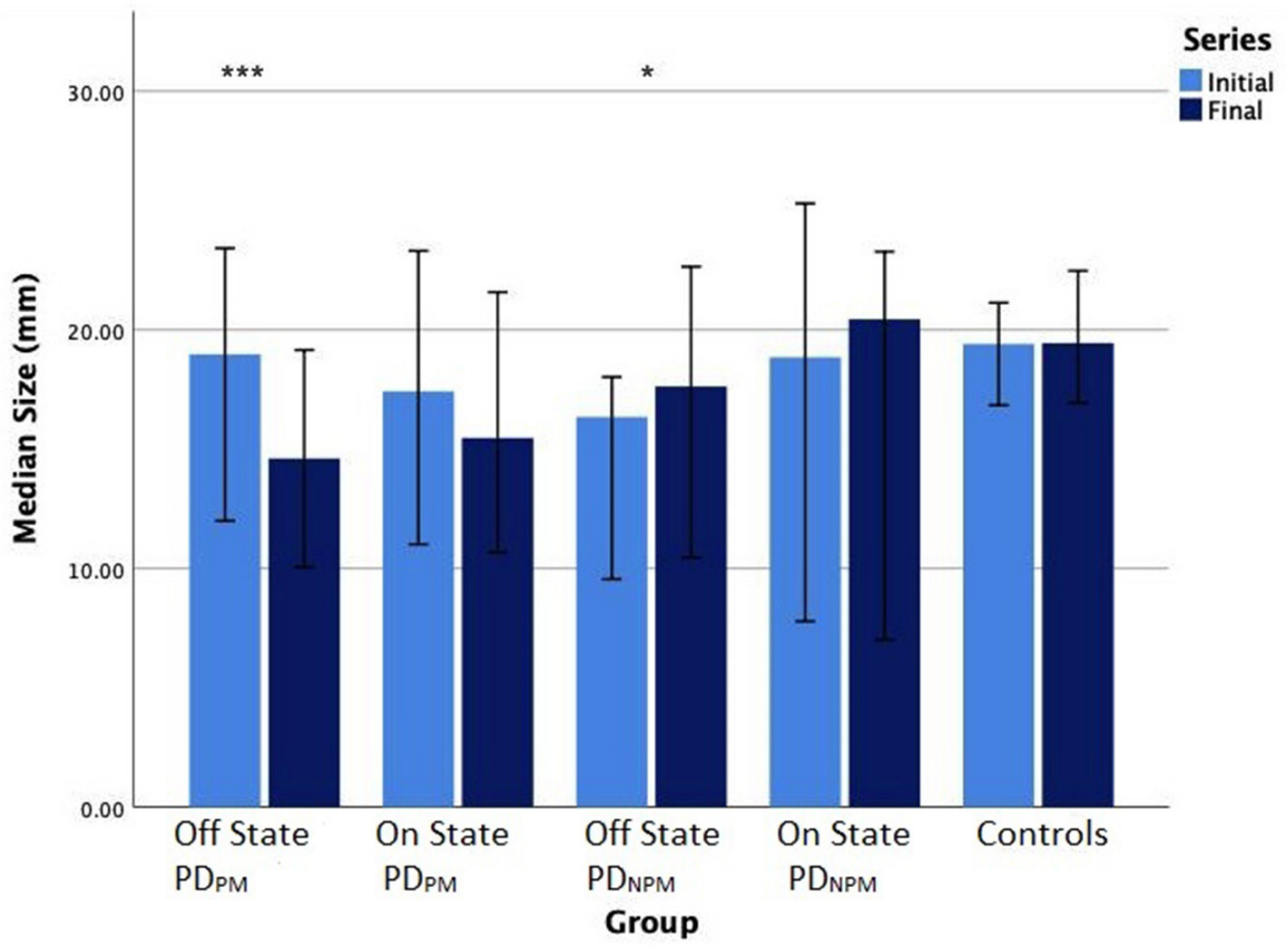
Bar chart (error bars for 95% confidence intervals) of median writing size (mm) of PD participants in off and on states, and controls. Wilcoxon signed-rank test significance levels: ***P < 0.001, *P < 0.05.

Sixteen PD participants showed a decrement >10% when off and were categorized as PD_PM_. Based on Wilcoxon test, initial and final sizes were significantly different (*p* < 0.001) in this group. This difference narrowed when on and was no longer significant. The remaining 8 PD participants composed the PD_NPM_ group. In PD_NPM_, there was a small increase in the size of the character e from initial to the final, which was significant in the off but not the on state (see Figure 2). Controls showed a slight increase in letter size from initial to final.

We found that control participants exhibited an increment in the speed of writing from left to right. PD_NPM_, though slower than controls, also increased their speed across the line of letters in their off state. With the PD_PM_ group, there was little change in speed between initial and the final letters.

PD_NPM_ scripts showed greater responses to levodopa than PD_PM_, and this effect was stronger for letter speed than size. Writing speeds were significantly greater for PD_NPM_ when on, and the increase in speed from the initial to final letters remained obvious. Smaller though significant speed increase was seen when on in the PD_PM_ group, but without speed change from initial to final letters.

Comparisons with control data in Table 4 indicate the relative magnitude of levodopa effects. Letter size for PD_NPM_ approached control values after levodopa, both for initial and final characters, with no significant differences. Speed, expressed in mm/sec, improved after levodopa in the PD_NPM_ group by 7.1 (initial letters) and 10.5 (final letters). The difference between on state PD_NPM_ and control writing speed for final letters also became insignificant (*p* = 0.26). The levodopa effect on speed in PD_PM_ was much smaller—only 3.1 (initial letters) and 3.6 (final letters)—with persisting differences from control values (*p* = 0.001).

**Table 4:** Size and speed of character writing, and pen-pressure, for different groups of PD and controls.

**Medication State**	**Series Initial-I Final-F**	**Size**		**Speed**		**Pressure**	
		**Median [IQR 25^**th**^-75^**th**^ percentile]**	***p***	**Median [IQR 25^**th**^-75^**th**^ percentile]**	***p***	**Median [IQR 25^**th**^-75^**th**^ percentile]**	***p***
**Progressive micrographia (PD**_**PM**_**)** ***N*** **=** **16**							
Off	I	18.97[12.45**–**23.10]	<0.001	20.70[11.95**–**30.17]	0.980	0.47[0.01**–**0.57]	0.034
	F	14.61[10.48**–**19.06]^🡻^		20.68[13.48**–**26.70]		0.41[0.01**–**0.54]	
On	I	17.42[11.40**–**23.15]	0.083	23.81[17.32**–**38.29]	0.562	0.48[0.01**–**0.68]	0.130
	F	15.47[10.75**–**21.25]		24.30[16.47**–**37.24]		0.43[0.01**–**0.67]	
**Non-progressive micrographia (PD**_**NPM**_**)** ***N*** **=** **8**							
Off	I	16.35[15.00**–**17.55]	0.039	19.03[10.75**–**24.47]	0.008	0.49[0.36**–**0.58]	0.945
	F	17.63[14.74**–**20.00]^🡹^		26.67[14.10**–**38.56]^🡹^		0.47[0.34**–**0.60]	
On	I	18.85[16.3**–**21.07]	0.313	26.12[14.79**–**37.26]	0.016	0.51[0.40**–**0.65]	0.195
	F	20.44[17.53**–**21.30]		37.16[16.74**–**49.90]^🡹^		0.50[0.34**–**0.59]	
**Controls**							
Controls	I	19.40[16.33**–**21.44]	0.13	38.76[26.51**–**46.91]	0.001	0.55[0.01**–**0.73]	0.790
	F	19.44[16.63**–**22.47]		41.23[29.25**–**58.01]^🡹^		0.52[0.01**–**0.71]	

*Two-tailed Wilcoxon signed-rank test was performed to compare initial and final series. IQR, interquartile range*.

The PD_PM_ group were not able to maintain pen-pressure from left to right. This produced a significant initial to final difference in the off state, with little improvement when on. Pen-pressure was more consistently applied in PD_NPM_ scripts, for both off and on.

Tables 4, 5 demonstrate that, overall, the decremental aspects of PD_PM_ obtained little levodopa benefit. Comparing initial and final letters, size and pen-pressure decrements remained, while the lack of left-to-right speed increment was not remedied.

**Table 5:** Mann-Whitney test^a^ and Wilcoxon test^b^ *p*-values for group differences, including medication effects.

**Groups**	**Speed (Initial)**	**Speed (Final)**	**Size (Initial)**	**Size (Final)**	**Pen-pressure (Initial)**	**Pen-pressure (Final)**
PD_NPM_-Off and Controls^a^	**0.001**	**0.041**	**0.029**	0.258	0.535	0.728
PD_NPM_-On and Controls^a^	**0.041**	0.258	0.815	0.931	0.815	0.896
PD_PM_-Off and Controls^a^	**0.001**	** <0.001**	0.890	**0.009**	0.456	0.307
PD_PM_-On and Controls^a^	**0.016**	**0.001**	0.508	0.064	0.804	0.699
PD_PM_-Off and PD_PM_-On^b^	**0.006**	**0.011**	0.234	0.255	0.159	0.464
PD_NPM_-Off and PD_NPM_-On^b^	**0.017**	0.069	0.069	0.208	0.208	0.401

*Significant p-values shown in bold type*.

As shown in Table 2, the MDS-UPDRS-III scale registered a significant response to levodopa (*p* < 0.05) for both groups of PD patients. The PD_NPM_ group improved from 23.9 to 18.9, an overall motor response score of 5.0, while for PD_PM_ the improvement was 8.2.

**Figure 3:**
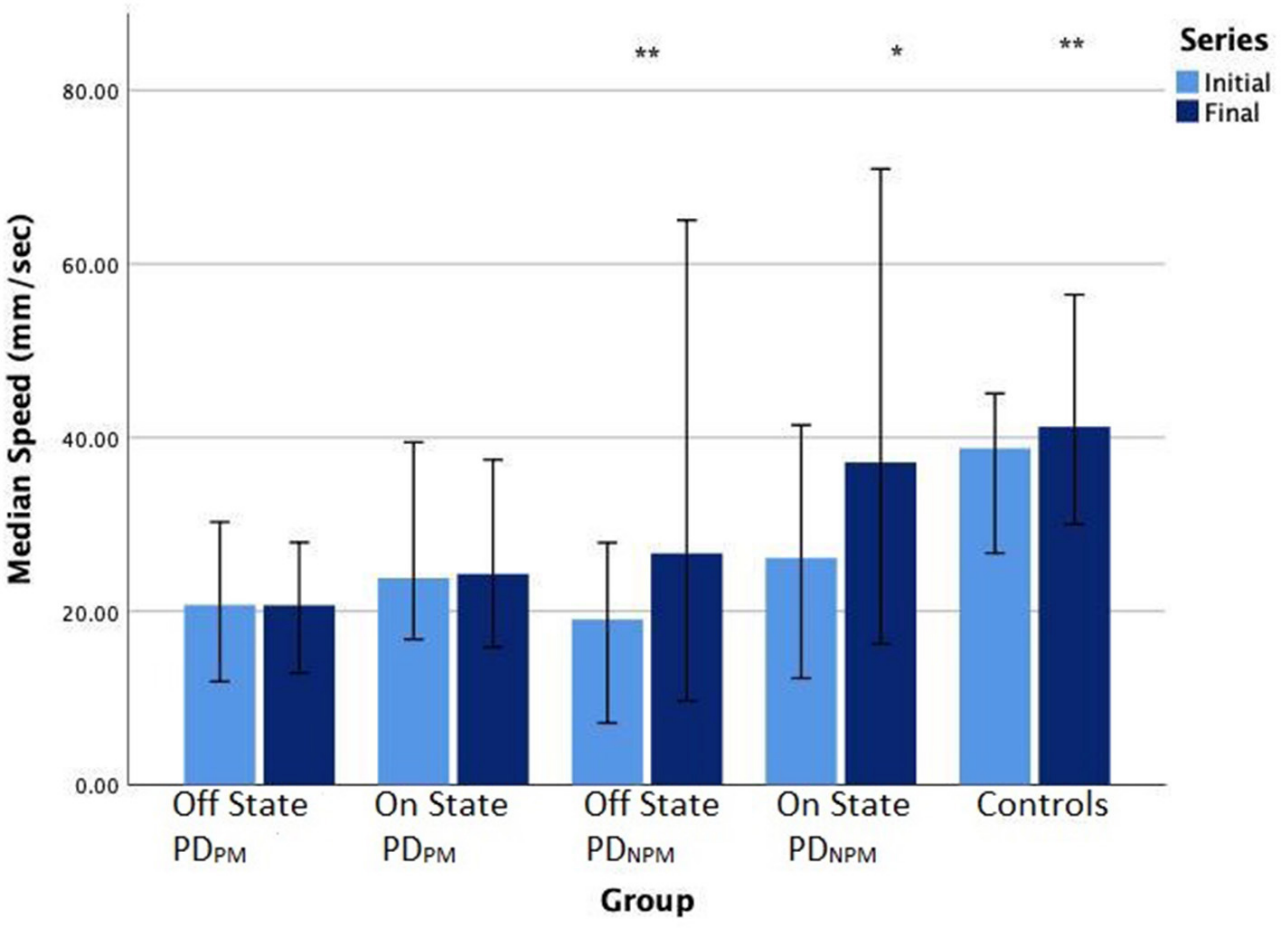
Bar chart (error bars for 95% confidence intervals) of median writing speeds (mm/sec) of PD participants in off and on states, and controls. Wilcoxon signed-rank test significance levels: **P < 0.01, *P < 0.05.

Table 4 displays the size and speed of writing of the character e for PD_PM_ and PD_NPM_ groups in off and on states, along with the control values. This data is embedded in Figures 2, 3. The group difference statistical analysis performed using Mann-Whitney tests is shown in Table 5.

## Discussion

Levodopa had significantly different effects on parkinsonian dysgraphia depending on whether or not progressive micrographia is present. PD_PM_ had less levodopa improvement to writing in general. Notably, the decremental features of progressive micrographia are relatively resistant to dopaminergic treatment. One observation about normal handwriting is important in understanding our findings. The control group showed an increase in speed from initial to final letters. This possibly reflects changes in muscle activation as wrist and elbow movement come increasingly into play when writing from left to right (25). The same tendency was present in PD_NPM_ (10). PD_PM_ lacks this left-to-right augmentation of writing speed.

The PD_NPM_ group had increases in both letter size and writing speed after levodopa that were greater than those seen in PD_PM_. In PD_NPM_ the greatest improvements in on state writing speed were seen in the final letter series, approaching the values of control left-to-right speed augmentation, and showing strong statistical significance. In PD_PM_, improvement in speed after levodopa was only half as much, and the blunting of left-to-right augmentation of writing speed largely persisted. The other aspect of motor decrement in PD_PM_, a fall-off in pen pressure with final letters, was similar before and after levodopa. Figure 1 provides a good illustration of the findings in PD_PM_. Some improvement in overall letter size is present after levodopa in this specimen, including the final characters. But waves of decrement are still obvious.

Our finding that the writing decrements of PD_PM_ show little responsiveness to levodopa broadly agrees with some previous research. Ling et al. (8) and Wu et al. (12) each used different definitions of progressive micrographia, and neither conducted kinematic measurements. Both reported that decrements in script size persisted in patients with PD despite levodopa therapy. Ling et al. (12) found a modest improvement in writing size in the on phases of their subjects. In Wu et al. (8) study, levodopa hardly changed character size when progressive micrographia was present, whereas there was significant improvement in a consistent micrographia group.

While we found progressive micrographia is generally less responsive to levodopa, this did not correspond to overall parkinsonian motor disability or its responsiveness to levodopa. The PD_PM_ group had a marginally higher MDS-UPDRS-III score. The motor response to levodopa was, however, a little better for PD_PM_ with an improvement of 8.0 compared with 5.3 for PD_NPM_. Dominant upper limb bradykinesia was slightly greater for PD_PM_, with a slightly smaller levodopa response. None of these differences were significant. Wu et al. (8) had also shown that progressive micrographia is not associated with a worse overall motor disability score, though comparisons of levodopa motor benefit were not given.

Bradykinesia is a shorthand for complex disturbances of initiation and execution of actions and the ability to sustain them. Slowness is not necessarily its most prominent component. Akinesia (failure to initiate movement) and hypokinesia (underactive movement) are part of bradykinesia, as is the sequence effect—repetitive movements becoming smaller or slower. Handwriting analysis has shown reduced speed and size in PD, and this has been used for the detection and monitoring of disease symptoms (22, 26, 27). The defining feature of progressive micrographia, though, represents the sequence effect. Research into other manifestations of the sequence effect mirrors our observations in handwriting. Kang et al. (28) using a pegboard task, found that levodopa improved overall speed but not decrement. Several studies have looked at bradykinesia in finger tapping (12, 29, 30). While a range of kinematic speed and rhythm measures are improved with levodopa, there is little change in decrement of amplitude.

Using abstract computerized methods, Gangadhar et al. (31) modeled progressive micrographia as a specific combination of moment-to-moment depletion of striatal dopamine and basal ganglia network dynamics. The sequence effect appears to be an integral component of bradykinesia. Parkinsonian movements are generally underscaled with respect to desirable range and speed, consistent with a defect in ‘motor energy'. Decrement, therefore, seems to reflect cumulative energy cost, (32) perhaps explaining why it has a poorer dopaminergic response than other kinematic or dimensional aspects of writing.

This study has shown some caveats on the use of handwriting analysis to monitor the dopaminergic responses of patients with PD. There are two patterns of dysgraphia in PD—one with progressive micrographia and one without. The significant differences in their handwriting response to levodopa appear unrelated to the overall motor benefit from the drug. While we are unable to conclude the mechanisms underpinning these differences, we recommend that the two should be distinguished in handwriting research because of their dissimilar pharmacological characteristics. Further studies need to investigate the energy costs of progressive micrographia. One possible explanation is that people with this form of parkinsonian dysgraphia are more impacted by sequence effects than those without.

## Limitations

The study sample size used here is similar to otherwise comparable studies, though not sufficient to fully investigate possible demographic factors. A defined off state and test-dose method is a standard way to evaluate the levodopa motor response. This method did, however, impose an unvarying order of off and on measurements. Another limitation is that effect of dosage was not assessed and hence it is not possible to comment on treatment options.

## Conclusion

The effect of levodopa on parkinsonian dysgraphia is reduced by the presence of progressive micrographia. The decrement of progressive micrographia is a manifestation of the sequence effect, the aspect of bradykinesia that is most resistant to dopaminergic treatment. Nevertheless, it does not denote that general motor disability is greater, or shows less response to levodopa. Computerized handwriting analysis has been proposed as a way of tracking the progression of PD and efficacy of antiparkinsonian treatment. By highlighting relationships between progressive micrographia and medication response, our findings imply that caution is needed in the interpretation of such measurements.

## Data Availability Statement

The raw data supporting the conclusions of this article will be made available by the authors, without undue reservation.

## Ethics Statement

The studies involving human participants were reviewed and approved by the study was conducted in accordance with the Helsinki Declaration on human experiments (revised 2004) and was approved by the Monash Health and RMIT University Human Research Ethics Committees. All participants in this study gave their written informed consent prior to data recording. The patients/participants provided their written informed consent to participate in this study.

## Author Contributions

PZ involved in conducting experiments, data analysis, drafting the article, software design and development, selection of analytical tools, statistical analysis, and literature review. DK involved in concept and design of work, selection of analytical tools, critical revision of the article, literature review, participated in manuscript preparation, and final approval of the version to be published. PK involved in clinical support, critical revision of the article, and participated in manuscript preparation. SP involved in statistical analysis, and review of the article. SR involved in clinical support, KW and KN involved in experimental support. All authors were involved in manuscript review.

## Conflict of Interest

PZ was employed by the company IBM Research Australia. The remaining authors declare that the research was conducted in the absence of any commercial or financial relationships that could be construed as a potential conflict of interest.
